# Dimethyl Derivatives of 2,2′-Bipyridine as Ligands in [W(CN)_6_(bpy)]^2−^-Type Complexes

**DOI:** 10.3390/molecules29020444

**Published:** 2024-01-16

**Authors:** Natalia Basta, Janusz Szklarzewicz, Maciej Hodorowicz, Anna Jurowska

**Affiliations:** Faculty of Chemistry, Jagiellonian University, Gronostajowa 2, 30-387 Kraków, Polandhodorowm@chemia.uj.edu.pl (M.H.); jurowska@chemia.uj.edu.pl (A.J.)

**Keywords:** tungsten, cyanido ligands, bipyridine, crystal structure, UV-Vis spectroscopy

## Abstract

The thermal decomposition of (XMebpyH)_3_(H_3_O)[W(CN)_8_]·3H_2_O (where XMebpy denotes 4Mebpy 4,4′-dimethyl-2,2′-bipyridine or 5Mebpy 5,5′-dimethyl-2,2′-bipyridine) in glycerol results in the coordination of XMebpy. Salts of the anion formula [W(CN)_6_(XMebpy)]^2−^ were isolated for PPh_4_^+^ and/or AsPh_4_^+^ cations as well as for K^+^, Rb^+^, and Cs^+^ ones. X-ray single-crystal analyses for tetraphenyl-phosphonium and tetraphenyl-arsonium cations are described. IR, UV-Vis, and cyclic voltammetry data are presented. The results were compared with those for [W(CN)_6_(bpy)]^2−^ (bpy = 2,2′-bipyridine) ion salts.

## 1. Introduction

Tungsten (IV) coordination compounds are an area of ongoing research, starting from the octacyanometallates to the currently described ion-based compounds [W(CN)_6_(bpy)]^2−/−^ [1,2,3,4,5,6,7,8,9,10,11,12]. Molybdenum, niobium, and tungsten octacyanometallates are interesting types of coordination compounds because systems can be built on their base multicores with cyanide bridges [13,14,15,16,17]. Their polymeric structure with small metal-to-metal distances contributes to the applicability of these systems in solar energy conversion, molecular electronics, research on magnetism and photomagnetism, and microporous materials with adsorption abilities [13,14,18,19,20]. Heteroleptic systems based on [W(CN)_6_(LL)]^n−^ ions (where n = 1 or 2) are being intensively explored due to the possibility of creating coordination polymers using bridging cyanido ligands [7]. Additionally, with intensive MLCT bands, these systems have more applications than systems containing only cyanido ligands. Unfortunately, only two such complexes are known—LL = bpy and 1,10-phenanthroline [2]. For the latter, only a synthetic procedure and some basic properties have been described. This is caused by the extremely low yield of synthesis resulting from its rigid ligand structure. No other complexes of this type are known. This is caused mainly by their synthesis, which requires the presence of a protonated organic precursor in the form of a cation in a [W(CN)_8_]^4−^ anion; moreover, the protonation constant of the precursor must be in a narrow range, the precursor must have two nitrogen atoms in the proper configuration for coordination, and the precursor must be flexible for stepwise nitrogen coordination, etc. Coordination compounds consisting of a [W(CN)_8_]^4−^ ion can also be used to form ion pairs with anion–cation charge transfer transitions (IPCTs) [21]. These types of relationships can potentially find applications in solar cells, catalytic reactions and optics, as well as energy storage and switching technologies. In this manuscript, we present the synthesis and properties of two new [W(CN)_6_(LL)]^2^—type complexes, synthesized with a relatively good yield (for this type of compound), leading to double the number of such anions.

## 2. Results and Discussion

### 2.1. General Remarks to Syntheses

**Figure 1:**
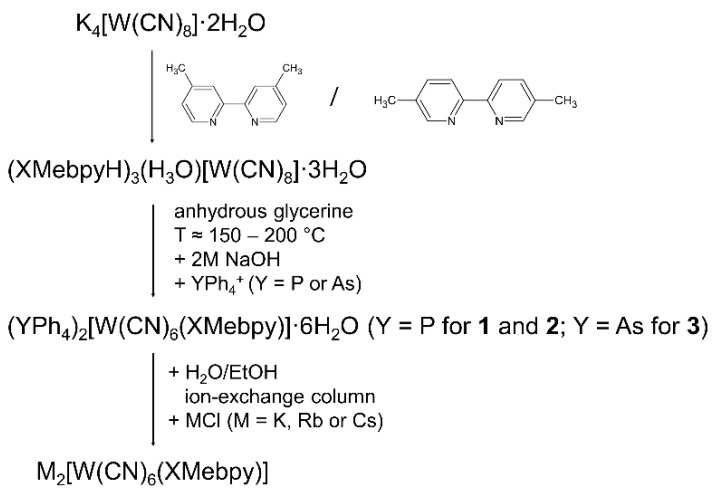
Synthesis scheme of [W(CN)_6_(XMebpy)]^2−^ complexes.

A multi-stage scheme for the synthesis of complexes with [W(CN)_6_(XMebpy)]^2−^ ions is shown in Figure 1.

As a result of the syntheses, a series of complexes based on [W(CN)_6_(XMebpy)]^2−^ ions were obtained. For the 4,4′-Mebpy ligand, a tetraphenylphosphonium salt as well as the salts of selected alkali metals were isolated. For the 5,5′-Mebpy ligand, an additional tetraphenylarsonium salt was obtained, and from this salt, a crystal suitable for X-ray structure analysis was received. Attempts were also made to obtain complexes with the 6,6′-Mebpy ligand, but during its synthesis, at the stage of heating over a burner flame, the formation of a light green solution was observed—not purple, as in the case of the other two compounds. The numerous trials under different experimental conditions had no effect. At this stage, the research related to this dimethyl derivative was discontinued, stating that the expected product was probably not formed due to steric hindrances—the close proximity of the methyl group to the nitrogen atoms of the pyridine ring and their strong interactions with cyanido ligands. The elemental analysis of compounds **1**–**3** is consistent with the data obtained from the X-ray structural analysis. For alkali metal salts only, IR spectra were obtained due to the small amount of the product, and the purpose of this was only to show that it is also possible to obtain this type of salt. All other measurements (CV, TG, and UV–Vis) were performed only for compounds **1** and **2** due to their having the highest quantity of pure product.

### 2.2. IR Spectra

**Figure 2:**
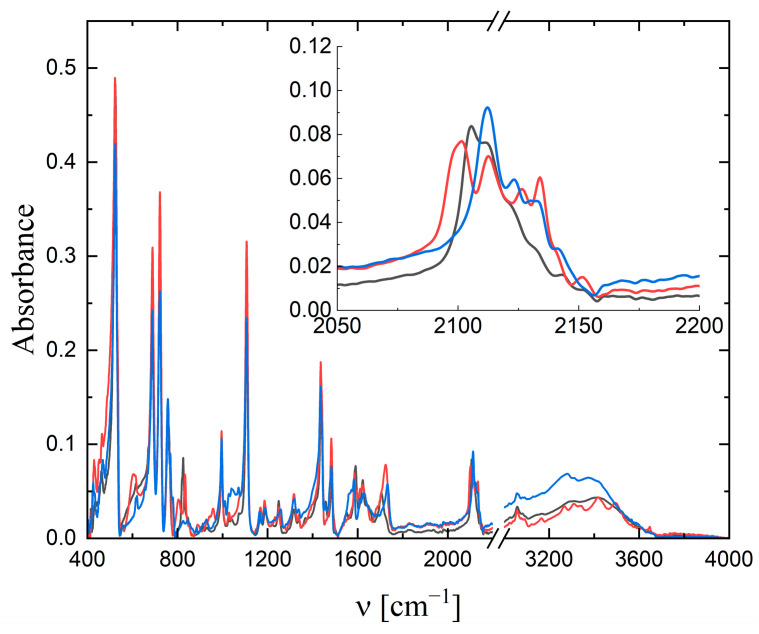
IR spectra of **1** (black line), **2** (red line), and (PPh_4_)_2_[W(CN)_6_(bpy)]·4H_2_O (blue line).

**Figure 3:**
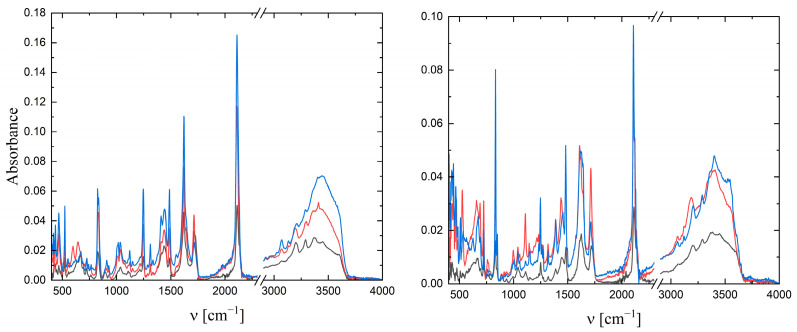
IR spectra of [W(CN)_6_(4,4′-Mebpy)]^2−^ (**left side**) and [W(CN)_6_(5,5′-Mebpy)]^2−^ (**right side**) with alkali metal salts. Black line for K^+^, red line for Cs^+^, and blue line for Rb^+^.

Figure 2 shows the IR spectra of **1** and **2** compared to (PPh_4_)_2_[W(CN)_6_(bpy)]·4H_2_O [22]. The spectra were very similar because they were dominated by the bands associated with the presence of the cation PPh_4_^+^, which had characteristic bands in the range of 680–760 cm^−1^ and an intense band at about 1100 cm^−1^. The spectra differed in the vibration range characteristic of cyanido ligands because of the different interactions present in the structures. The presence of methyl substituents in the bpy ligand (for **1** and **2**) also caused some changes in the spectra—for example, at 830 and 1250 cm^−1^. In Figure 3, the IR spectra of the alkali metal salts of [W(CN)_6_(XMebpy)]^2−^ are presented. For [W(CN)_6_(4,4′-Mebpy)]^2−^, bands characteristic of cyanido ligands were observed at 2112 and 2138 cm^−1^ (for the K^+^ salt), at 2108 and 2141 cm^−1^ (for the Cs^+^ salt), and at 2110 cm^−1^ (for the Rb^+^ salt), while for [W(CN)_6_(4,4′-Mebpy)]^2−^, these bands were located at 2110 and 2137 cm^−1^ (for K^+^), at 2106 and 2135 cm^−1^ (for Cs^+^), and at 2107 and 2136 cm^−1^ (for Rb^+^). For alkali metal salts with [W(CN)_6_(bpy)]^2−^ anions, which have been previously studied, up to five bands in this range were observed—for example, for rubidium salt at 2112, 2119, 2130, 2144, and 2149 cm^−1^ [7]. The lowest energy bands were assigned in these salts, to cyanido ligands not involved in interactions with cations; thus, it seems that the network of intermolecular interactions is definitely poorer in the case of a 4,4′-Mebpy and 5,5′-Mebpy ligands, which may be related to the presence of methyl substituents. Solving this problem will require the determination of the crystal structure salts with alkaline cations.

### 2.3. UV-Vis Spectra

**Figure 4:**
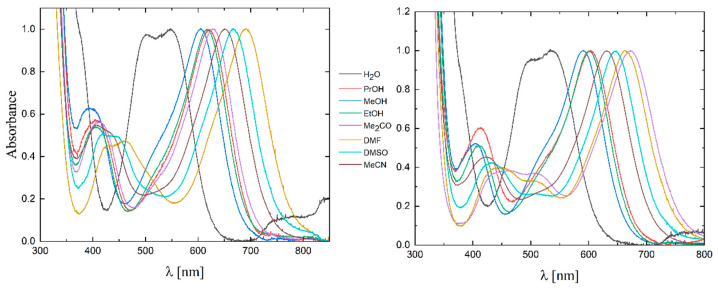
Qualitative UV–Vis spectra for **1** and **2** in different solvents; d = 1 cm.

For complexes **1** and **2**, the UV–Vis spectra in water and different organic solvents were measured (Figure 4). The spectra were normalized to an absorbance equal to 1.0 so that the intensity of the lowest-energy MLCT band was identical in all solvents tested. In Table 1, the color of the solution, the wavelength at which the absorption maximum was observed, and the Reichardt parameter [23,24] are listed.

**Figure 5:**
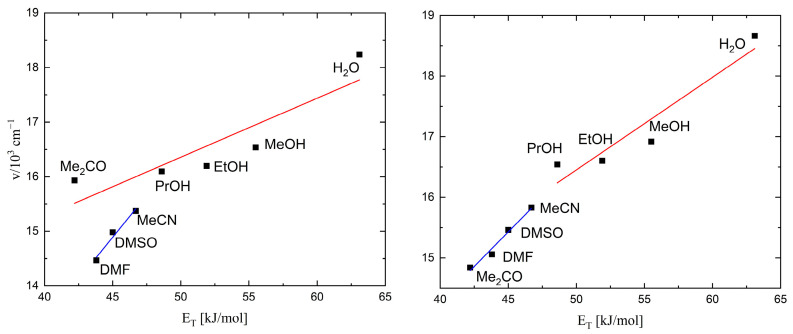
Dependence of the transition energy on the Reichardt parameter for **1** (**left side**) and **2** (**right side**).

A graph showing the solvatochromic effect is presented in Figure 5. For complex **1** (left side in Figure 5), there are two curves: one for polar solvents such as distilled water and several kinds of alcohols (red curve), and one for non-hydroxyl (non-polar) solvents such as acetone, DMF, DMSO, and MeCN (blue curve). As a result of the much larger polarity of water molecules, the water was quite far away from the other data points on the diagram, slightly disturbing the appearance of the curve for the polar solvents. There was an unusual situation with the acetone, which belongs to the non-polar solvents but was located on the polar solvents’ curve; this indicates the specific effects of this solvent on the ligands of the complex ion. The presence of two correlation lines for different types of solvents indicates the specificity of their interaction with the [W(CN)_6_(4,4′-Mebpy)]^2−^ anion. For complex **2** (right side in Figure 5), unlike the previously described salt with the 4,4′-Mebpy ligand, the position of the band for the acetone was on the correlation line of the non-hydroxylic solvents.

**Figure 6:**
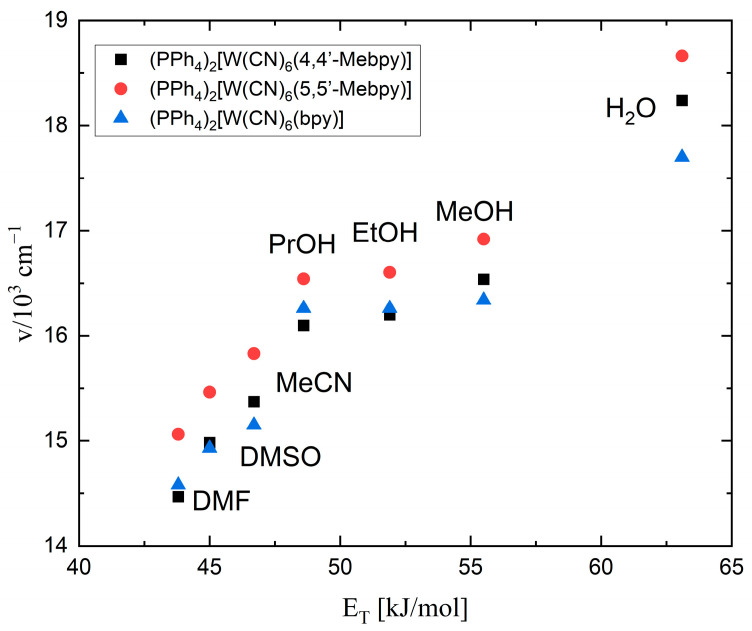
Dependence of the MLCT band position on the Reichardt E_T_ parameter for **1**, **2,** and (PPh_4_)_2_[W(CN)_6_(bpy)]·4H_2_O [22,25].

The results obtained were also summarized in one graph together with the data for the (PPh_4_)_2_[W(CN)_6_(bpy)]·4H_2_O salt [22,25] (Figure 6). There were slight shifts of some of the points here, illustrating the impact of methyl groups on the energy of MLCT transitions. The location of the bands for complex **1** was more similar to the value of the compound with the 2,2′-bpy ligand. In the case of salt **2**, the course of the curves was the most regular, but had a different slope compared to the other salts. As can be seen from the presented data, the most energetic MLCT bands were seen for **2**, while for **1** and for (PPh_4_)_2_[W(CN)_6_(bpy)]·4H_2_O, they had lower energies and had very similar transition energies. In general, the introduction of methyl substituents in bpy ligands increased the energy of the MLCT band compared to the (PPh_4_)_2_[W(CN)_6_(bpy)]·4H_2_O [22,25]. These energies increased by 2.50 kJ/mol for **1** and by 8.03 kJ/mol for **2** in MeCN. This is a very significant increase in energy, indicating the influence of the CH_3_ group on the electron acceptor properties of the nitrogen atom properties due to its para position in **1**, decreasing the positive charge on the nitrogen and thus increasing the MLCT transition energy, while in **2,** the meta position of the CH_3_ versus the nitrogen atom was responsible for the much higher energy of the MLCT transition. 

### 2.4. Cyclic Voltammetry Measurements

**Figure 7:**
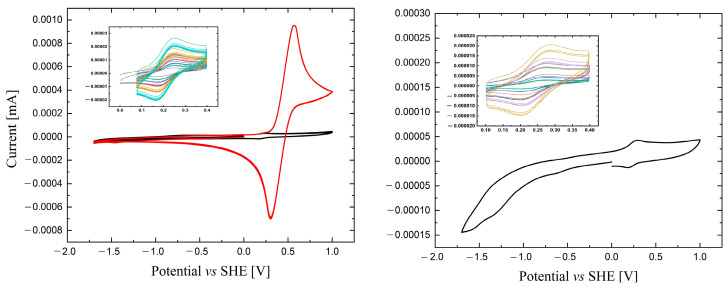
Cyclic voltammogram for compound **1** (**left side**) and **2** (**right side**) in 0.1 M acetonitrile solution; Bu_4_NPF_6_ was used as the electrolyte. Red line (**left side**) shows the voltammogram after ferrocene addition.

Cyclic voltammetry measurements were performed for **1** and **2** (Figure 7). On the left side of Figure 7, the red line shows the voltammogram of **1** after the addition of ferrocene, while the black line shows the voltammogram in the entire range for complex **1**. The inset in the upper right corner presents changes at different scan speeds. Compound **1** shows a reversible redox potential with a value of 0.179 V. Comparing the potential value with the data for a (PPh_4_)_2_[W(CN)_6_(bpy)]·4H_2_O described in the literature (E = 0.292 V) [26], it was concluded that the redox potential of the compound based on the 4,4′-Mebpy ligand was much lower. Complex **2** (right side in Figure 7) showed a reversible redox potential, with a value of 0.215 V; this potential was also lower than for the analogous salt with 2,2′-bpy, but it had a higher value compared to the salt with the 4,4′-Mebpy.

### 2.5. Thermogravimetric Analysis

**Figure 8:**
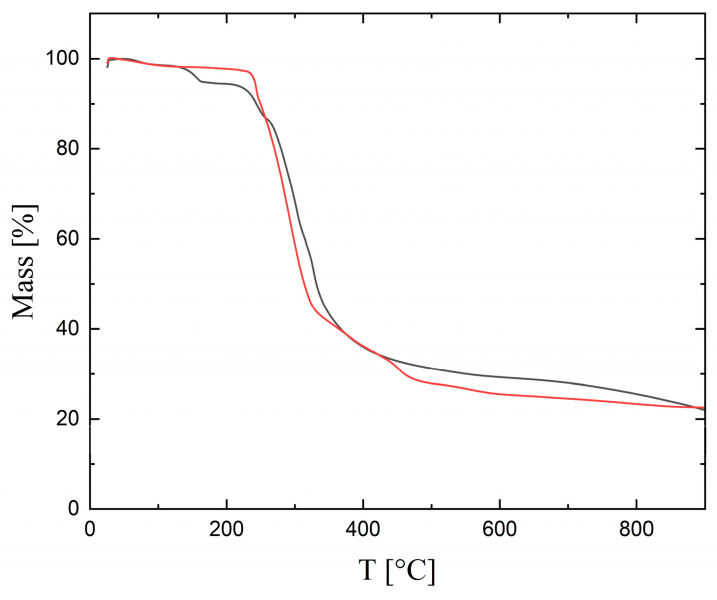
TG for **1** (black line) and **2** (red line). Scan speed 10°/min, Ar.

For complex **1** and **2**, the thermogravimetric analysis was performed in the 20–900 °C range. Both complexes (see Figure 8) had a similar decomposition pathway; in total, the same weight loss occurred at the end. The compounds were stable up to a temperature of approx. 250 °C. The initial mass losses were related to the departure of hydration water molecules. In the case of complex **1**, it is possible to observe the release of three and a half water molecules, initially at a temperature of about 126 °C (loss of experimental mass: 4.82%, calculated: 4.83%). Next, the decomposition of the complex ion showed a well-marked peak of departure for the remaining water molecules and the HCN (the experimental mass loss for the release of six H_2_O and one and a half of HCN molecules was 11.8%, calculated 11.4%). For compound **2**, a slight decrease of about 1.5% in the initial mass was observed at a temperature of about 50 °C, which corresponds to the release of the crystallization water. At temperatures above 230 °C, there was a sharp decrease in mass, which was associated with the decomposition of the main product based on the 5,5′-Mebpy ligand. In the case of compound **2**, the water molecules were more strongly bound in the structure, and therefore they did not leave as easily as in the case of complex **1**.

### 2.6. Crystal Structure of ***1*** and ***3***

**Figure 9:**
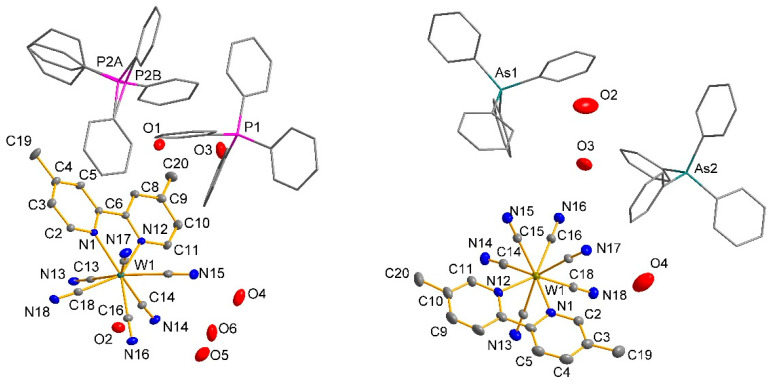
Asymmetric part of the unit cell of compound **1** (**left**) and compound **3** (**right**) with the adopted numbering schemes. The figures show the environment of local coordination for the [W(CN)_6_(bpy)]^2−^ and PPh_4_^+^ and AsPh_4_^+^ cations. The cations molecules are shown in a simplified diagram for clarity. For clarity in the figure, all H atoms have also been omitted. All non-hydrogen atoms are represented at 30% probability thermal ellipsoids.

The asymmetric parts of the crystal unit cells of all assemblies with the adopted numbering schemes are shown in Figure 9. In Table 2, the crystallographic data and detailed information on the structure solution and refinement are presented.

**Figure 10:**
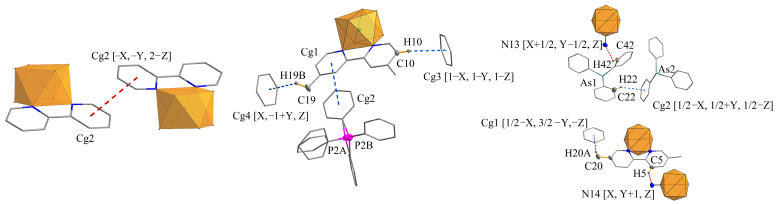
Comparison of structural motifs for **2,2′-bpyPPh_4_ [27]**, **1,** and **3**, respectively, resulting from the presence of the π⋯π and C–H⋯π-type fundamental interactions. Some atoms were omitted for clarity.

**Figure 11:**
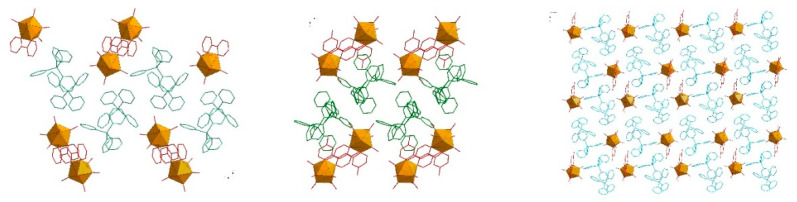
Comparison of the packing of three structures: **2,2′-bpyPPh_4_** [27] (**left**), **1** (**middle**), and **3** (**right**). The layers containing PPh_4_^+^ and AsPh_4_^+^ cation molecules are marked in green and blue, respectively. The coordination polyhedra of W atoms are marked in yellow, and the ligands around these atoms are marked in red. Hydrogen and oxygen atoms have been removed for clarity.

The asymmetric elemental cell parts of structures **1** and **3** had very similar contents and include the coordination anion [W(CN)_6_(bpy)]^2−^, which in the case of **1** contained a bipyridyl ligand (bpy) substituted with CH_3_- groups at position 4,4′; in the case of structure **3**, the bpy ligand was substituted at the 5,5′ positions. The composition was completed by two mono-positive PPh_4_^+^ cations in the case of structure **1** and AsPh_4_^+^ in the case of **3**. The whole was completed by water molecules, of which there were six in the case of **1**; in the case of compound structure **3** there were three water molecules, except that one had an occupancy of 0.5 (Figure 9). The parameters of selected bonds and the valence angles for structures **1** (4,4′-Mebpy) and **3** (5,5′-Mebpy) compared to the literature structures of (PPh_4_)_2_[W(CN)_6_(bpy)]·4H_2_O (**2,2′-bpyPPh_4_**) [27] and (AsPh_4_)_2_[W(CN)_6_(bpy)]·3.5H_2_O (**2,2′-bpyAsPh_4_**) [28] are presented in Table 3 and Table 4. In both structures, the bond lengths in the coordination sphere of the tungsten atom had values close to each other and were consistent with the literature data [27,28]; however, the average length of the W–C and W–N_bpy_ bonds was longer for the dimethyl derivatives (**1** and **3**) than for complexes with an unsubstituted ligand 2,2′-bpy. This indicates that the presence of methyl substituents in the pyridine ring contributed to the weakening (lengthening) of the tungsten–ligand bond, and this weakening was slightly greater in the case of the 5,5′-Mebpy ligand (**3**). The volumes of the unit cells of all the compounds tested differed significantly from each other, and the structure of compound **3** had the largest volume of up to 11,915.93(5) Å^3^. In structures **1** and **3**, whose bidentate ligand bpy contained methyl substituents, a decrease in crystal density was observed; the density for compound **1** had the lowest value of 1.409 Mg/m^3^. It is noteworthy that in structures **1** and **3**, the predominant intermolecular interactions were C–H⋯N, C–H⋯O-type hydrogen interactions, while the literature structures **2,2′-bpyPPh_4_** and **2,2′-bpyAsPh_4_** are characterized by the presence of classical O–H⋯O-type interactions (water molecule interactions) (Figure 10). An additional feature of these structures is the presence of π⋯π-type interactions involving the 2,2′-bpy ligand (Table 4). Characteristically, in cases **1** and **3**, only one interaction of this type could be observed (for **1**), while the dominant role in stabilizing the structure—omitting, of course, the typical hydrogen interactions—is played by C-H⋯π-type interactions (Table 5), in which PPh_4_^+^ and AsPh_4_^+^ cations are primarily involved. In addition, in the case of **2,2′-bpyPPh_4_** and **2,2′-bpyAsPh_4_**, a large bending of the 2,2′-bpy ligand molecule is observed, averaging 9.61° (Table 6). The presence of CH_3_- substituents in the 2,2′-bpy ligands causes the value of the molecular bending to decrease drastically, and the molecular bending angles of the 4,4′-Mebpy and 5,5′-Mebpy ligands in **1** and **3** were 6.80° and 6.20°, respectively (Table 6). At the same time, with the bending of the 2,2′-bpy ligand molecules, the distance of the central W atom with respect to the plane determined by the nitrogen and carbon atoms of the bipyridyl ligand increased (Table 6). In structures containing substituted derivatives of the 2,2′-bpy ligand (**1** and **3**), an increase in the volume of the coordination polyhedron around the W atom was also observed, which is an obvious consequence of the decreasing influence of the deforming stresses of the bpy ligand molecules. In this context, it can be unequivocally stated that the presence of CH_3_- substituents in the rings of the 2,2′-bpy ligand resulted in a significant increase in the spatial occupation of these ligands, and thus, a much lower involvement of these ligands in π⋯π and C–H⋯π-type interactions with cations; this is expressed by the data in Table 4 and Table 5. Although each of the compounds analyzed crystallized in a different space group, the spatial arrangement of the cations and anions was similar and could be described as layered, with the layers resembling a zigzag or wave shape (see Figure 11). The distances between the layers for the central atoms (W···W) were similar, with values around 14 Å. The smallest distances between W···W atoms were observed in the structure of compound **1**, while the largest analogous distances characterized structure **3** (Table 7).

## 3. Experimental

### 3.1. Materials and Methods

(XMebpyH)_3_(H_3_O)[W(CN)_8_]·3H_2_O and (PPh_4_)_2_[W(CN)_6_(bpy)]·4H_2_O (**2,2′-bpyPPh_4_**) were prepared as described earlier [12,21,27]. All other materials and solvents were of analytical grade (Sigma-Aldrich, Saint Louis, USA) and were used without further purification. The IR spectra were recorded on a Shimadzu IRAffinity-1S equipped with a QATR 10 ATR (Shimadzu Corp., Kyoto, Japan) Microanalysis of carbon, hydrogen, and nitrogen was performed by means of an Elementar Vario MICRO Cube elemental analyzer (Elementar, Langenselbold, Germany). Electronic absorption spectra were recorded with a Shimadzu UV-3600 UV–VIS-NIR spectrophotometer (Shimadzu Corp., Kyoto, Japan) equipped with a CPS-240 temperature controller. Thermogravimetric measurements were performed on a TGA/SDTA 851e Mettler Toledo Microthermogravimeter (Mettler Toledo, Greifensee, Switzerland) in air or under an argon atmosphere with a scan speed 10 °C/min. The cyclic voltammetry measurements were carried out in DMSO with (Bu_4_N)PF_6_ (0.1 M) as the supporting electrolyte, using Pt as working and counter and Ag/AgCl as reference electrodes on an AUTOLAB/PGSTAT 128 N Potentiostat/Galvanostat (Metrohm, Herisau, Switzerland). E_1/2_ values were calculated from the average anodic and cathodic peak potentials: E_1/2_ = 0.5(E_a_ + E_c_). The redox potentials were calibrated versus ferrocene (0.404 V versus SHE), which was used as an internal potential standard for measurements in organic solvents to avoid the influence of a liquid junction potential; the final values are reported versus the standard hydrogen electrode (SHE).

### 3.2. Syntheses

In general, the procedure was analogous to that described previously [12].

#### 3.2.1. (PPh_4_)_2_[W(CN)_6_(4,4′-Mebpy)]·6H_2_O (**1**)

The (4,4′-MebpyH)_3_(H_3_O)[W(CN)_8_]·3H_2_O (0.50 g) was placed in a porcelain crucible and a small amount of anhydrous glycerin (4 drops, ca 0.2 mL) was added. The mixture was heated over a burner flame until the violet color of the resulting solution was observed. The resulting solution was added to a small amount of distilled water (ca 20 mL) with the addition of 4 mL of 2M NaOH. The resulting solution was filtered on a paper filter to eliminate the excess 4,4′-Mebpy, resulting in a clear purple solution. A stoichiometric amount of solid PPh_4_Cl was added to the solution and then the resulting dark purple precipitate was filtered and dried in air. Yield: 18.07%. Calcd. for C_66_H_64_N_8_O_6_P_2_W: C, 60.46; N, 8.55; H, 4.92%. Found: C, 60.92; N, 8.46; H, 4.72%.

#### 3.2.2. (PPh_4_)_2_[W(CN)_6_(5,5′-Mebpy)]·6H_2_O (**2**)

The procedure was analogous to that for **1**, except (5,5′-MebpyH)_3_(H_3_O)[W(CN)_8_]·3H_2_O (0.50 g) was used. The complex obtained was recrystallized by slow evaporation using methyl alcohol as a solvent, resulting in dark purple crystals. Yield: 13.24%. Calcd. for C_66_H_64_N_8_O_6_P_2_W: C, 60.46; N, 8.55; H, 4.92% Found: C, 60.47; N, 8.26; H, 4.34%.

#### 3.2.3. (AsPh_4_)_2_[W(CN)_6_(5,5′-Mebpy)]·5H_2_O (**3**)

The procedure was analogous to that used for **2**, but AsPh_4_Cl was used instead of PPh_4_Cl. A very small number of crystals were obtained, which was only sufficient for X-ray single crystal structure measurements and for elemental analysis. Calcd. for C_66_H_62_N_8_O_5_As_2_W·H_2_O: C, 56.66; N, 8.01; H, 4.61%. Found: C, 56.73; N, 8.14; H, 4.28%.

#### 3.2.4. Syntheses of M_2_[W(CN)_6_(XMebpy)] Salts, Where (M = K, Rb, Cs)

0.10 g of (PPh_4_)_2_[W(CN)_6_(XMebpy)] was dissolved in a small amount of a mixture of H_2_O–EtOH (1:1 volume ratio). The resulting violet solution was placed in the ion-exchange column, in hydrogen form, and the process of replacing the PPh_4_^+^ cations with the H^+^ cations was carried out, washing the column with distilled water. The darkest violet fraction was collected, and the excess of the respective chloride (KCl, RbCl, or CsCl) was added to it, and the solution was left for crystallization. After a few days, the products were filtered off and dried in air.

### 3.3. Crystallographic Data Collection and Structure Refinement

The diffraction intensity data for a single crystal of the new compounds were collected at 130(2) and 270(2) K for **1** and **3**, respectively, on a Rigaku XtaLAB Synergy-S diffractometer with mirror-monochromated Mo Kα radiation (λ = 0.71073 Å) for **1** and Cu Kα radiation (λ = 1.54184 Å) for **3**. Cell refinement and data reduction were performed using firmware [29]. The positions of all non-hydrogen atoms were determined using SHELXT software Version 2014/4 [30,31]. All non-hydrogen atoms were anisotropically refined using weighted full-matrix least-squares on F^2^. Refinement and further calculations were carried out using SHELXL-2018/3 [30,31]. All hydrogen atoms joined to carbon atoms were positioned with idealized geometries and refined using a riding model with Uiso(H) fixed at 1.2 Ueq (Carom). In the structure of compound **1**, one of the phosphorus atoms (P2), constituting the central atom of one of the two PPh_4_^+^ cations, shows the features of a slight positional disorder, and its positions in individual positions were in a mutual ratio 0.49:0.51(P2A:P2B). In both tested structures, a strong disorder of water molecules was also observed, and due to the impossibility of reasonably modeling this disorder, it was decided to use the SQUEEZE procedure in the PLATON software (Version 2023.1) [32]. Removing the electron density in this way, which represents one water molecule in both structures, does not substantially affect the overall model of either of the structures. Figures were made using Diamond ver. 4.6.1 software [33]. CCDC 2301687 and 2301692 contain the supplementary crystallographic data for **1** and **3**, respectively. These data can be obtained free of charge from The Cambridge Crystallographic Data Centre via www.ccdc.cam.ac.uk/data_request/cif (accessed on 11 January 2024).

## 4. Conclusions

The incorporation of weak electron-donating methyl substituents in the 2,2′-bipyridine ligand in [W(CN)_6_(bpy)]^2−^ anion salts results in multiple changes: in the energy of the MLCT band, its redox properties, its IR spectra, as well as the parameters of its X-ray crystal structure. The new complexes described are very important for studies related to heteroleptic [W(CN)_6_(bpy)]^2−^ anion salts. Now, for the first time, it will be possible to have three different anions of this type. The presence of methyl substituents (thus, steric hindrances) will allow us to study changes in packing schemes and to modify the intermolecular interactions and other properties of polymeric systems with (-W-CN-Me-)-type linear chains (where Me denotes any metal cation). The possibility of the structural modification of such systems plays a crucial role in the material sciences and in the synthesis of advanced materials, including new ferromagnetic materials.

## Figures and Tables

**Table 1:** UV–Vis data for MLCT band in **1** and **2**.

Solvent	Reichardt Parameter E_T_ (kJ/mol)	Solution Color	Wavelength (nm)	Solution Color	Wavelength (nm)
		1		2	
H_2_O	63.1	Violet	548	Violet	536
PrOH	48.6	Blue	621	Blue	605
MeOH	55.5	Blue	605	Blue	591
EtOH	51.9	Blue	617	Blue	602
Me_2_CO	42.2	Green	628	Green	674
DMF	43.8	Green	691	Green	664
DMSO	45.0	Green	667	Green	647
MeCN	46.7	Green	651	Green	632

**Table 2:** Crystal data and structure refinement for **1** and **3**.

		1 (4,4′-Mebpy)	3 (5,5′-Mebpy)
Empirical formula		C_66_H_56_N_8_O_6_P_2_W	C_132_H_104_As_4_N_16_O_5_W_2_
Formula weight		1302.97	2661.69
Temperature [K]		130(2)	270(2)
Wavelength [Å]		0.71073	1.54184
Crystal system		Triclinic	Monoclinic
Space group		*P* $\bar{1}$	*C* 2/c
Unit cell dimensions	*a* (Å)	13.3722(3)	30.70410(10)
	*b* (Å)	14.5777(3)	9.830
	*c* (Å)	16.8548(3)	39.49840(10)
	*α* (°)	73.537(2)	90
	*β* (°)	88.941(2)	91.74
	*γ* (°)	77.430(2)	90
Volume (Ǻ^3^)		3072.11(11)	11,915.93(5)
Z		2	4
Density (calculated) (g/cm^3^)		1.409	1.484
Absorption coefficient (mm^−1^)		1.990	5.236
F(000)		1320	5312
Crystal size (mm^3^)		0.200 × 0.100 × 0.100	0.200 × 0.100 × 0.100
Theta range for data collection (^o^)		2.457 to 30.719	2.880 to 80.534
Index ranges		−18 ≤ h ≤ 17 −19 ≤ k ≤ 20 −22 ≤ l ≤ 21	−39 ≤ h ≤ 39 −12 ≤ k ≤ 10 −50 ≤ l ≤ 50
Reflections collected		98,339	195,223
Independent reflections		16,322 (R(int) = 0.0509)	12,856 (R(int) = 0.0753)
Completeness to theta		99.8%	99.9%
Refinement method		Full-matrix least-squares on F^2^	Full-matrix least-squares on F^2^
Data/restraints/parameters		16,322/3/812	12,856/0/719
Goodness-of-fit on F^2^		1.063	1.051
Final R indices (I > 2σ(I))		R1 = 0.0346, wR2 = 0.0798	R1 = 0.0324, wR2 = 0.0841
R indices (all data)		R1 = 0.0407, wR2 = 0.0820	R1 = 0.0341, wR2 = 0.0852
Largest diff. peak and hole (e/Ǻ^3^)		1.314 and −1.268	1.008 and −1.343

**Table 3:** Bond lengths for (PPh_4_)_2_[W(CN)_6_(bpy)]·4H_2_O (described as **2,2′-bpyPPh_4_**), **1** (described as 4,4′-Mebpy), (AsPh_4_)_2_[W(CN)_6_(bpy)]·3.5H_2_O (described as **2,2′-bpyAsPh_4_**), and **3** (described as 5,5′-Mebpy).

2,2′-bpyPPh_4_ [27]		1 (4,4′-Mebpy)		2,2′-bpyAsPh_4_ [28]		3 (5,5′-Mebpy)	
Bond	Distance (Å)	Bond	Distance (Å)	Bond	Distance (Å)	Bond	Distance (Å)
W(1)-C(69)	2.147(3)	W(1)-C(14)	2.149(3)	W(1)-C(3)	2.139(5)	W(1)-C(14)	2.147(3)
W(1)-C(69)	2.148(3)	W(1)-C(18)	2.148(3)	W(1)-C(4)	2.146(5)	W(1)-C(18)	2.152(3)
W(1)-C(63)	2.154(3)	W(1)-C(17)	2.164(3)	W(1)-C(2)	2.147(5)	W(1)-C(17)	2.160(3)
W(1)-C(73)	2.159(3)	W(1)-C(16)	2.153(3)	W(1)-C(6)	2.155(5)	W(1)-C(16)	2.160(3)
W(1)-C(75)	2.163(3)	W(1)-C(13)	2.165(3)	W(1)-C(5)	2.156(5)	W(1)-C(13)	2.170(3)
W(1)-C(65)	2.164(3)	W(1)-C(15)	2.160(3)	W(1)-C(1)	2.157(5)	W(1)-C(15)	2.177(3)
W(1)-N(48)	2.215(2)	W(1)-N(12)	2.224(2)	W(1)-N(8)	2.206(4)	W(1)-N(12)	2.225(2)
W(1)-N(59)	2.225(2)	W(1)-N(1)	2.215(2)	W(1)-N(7)	2.208(4)	W(1)-N(1)	2.237(2)

**Table 4:** π···π interaction geometry [Å] for structures **1** and **3** with respect to structures **2,2′-bpyPPh_4_** and **2,2′-bpyAsPh_4_ [27,28]**.

	π^…^π	d(π^…^π)	Shift	Description
**2,2′-bpyPPh_4_**	Cg1…Cg1 [2 − X, 1 − Y, 2 − Z]	3.893(2)	1.349	Cg1: C91-C92-C93-C94-C95-C96 (PPh_4_^+^ cation) Cg2: N7-C8-C9-C10-C11-C12 (bpy ligand)
	Cg2…Cg2 [−X, −Y, 2 − Z]	3.8976(18)	1.889	
**1**	Cg1…Cg2	3.8875(1)	1.106	Cg1: N1-C2-C3-C4-C5-C6 (bpy ligand) Cg2: C45-C46-C47-C48-C49-C50 (PPh_4_^+^ cation)
**2,2′-bpyAsPh_4_**	Cg1…Cg1 [1 − X, −Y, 1 − Z]	3.806(3)	1.274	Cg1: C29-C30-C31-C32-C33-C34 (AsPh_4_^+^ cation)
	Cg2…Cg2 [2 − X, −Y, 2 − Z]	3.965(3)	1.620	Cg2: N7-C7-C8-C9-C10- C11 (bpy ligand)
	Cg3…Cg3 [1 − X, −Y, 2 − Z]	3.806(3)	1.800	Cg3: N8-C12-C13-C14-C15- C16 (bpy ligand)
**3**	no π⋯π-type interactions			

**Table 5:** C–H⋯π interaction geometry [Å, °] for structures **1** and **3** with respect to structures **2,2′-bpyPPh_4_** and **2,2′-bpyAsPh_4_** [27,28].

	C-H^…^π	H^…^Cg	X-H···Cg	X···Cg	Description
**2,2′-bpyPPh_4_**	C94-H94…Cg3 [2 − X, 1 − Y, 2 − Z]	2.96	158	3.840(5)	Cg3: C71-C72-C73-C74-C75-C76 (PPh_4_^+^ cation)
**1**	C10-H10…Cg3 [1 − X, 1 − Y, 1 − Z]	2.97	156	3.8627(1)	Cg3: C33-C34-C35-C36-C37-C38 (PPh_4_^+^ cation) Cg4: C21-C22-C23-C24-C25-C26 (PPh_4_^+^ cation)
	C19-H19B…Cg4 [X, −1 + Y, Z]	2.86	132	3.5884(1)	
**2,2′-bpyAsPh_4_**	C20-H11…Cg4 [1 + X, Y, Z]	2.87	134	3.591(7)	Cg4: C53-C54-C55-C56-C57-C58 (AsPh_4_^+^ cation)
	C32-H21…Cg5 [1 − X, −Y, 1 − Z]	2.81	157	3.698(6)	Cg5: C23-C24-C25-C26-C27-C28 (AsPh_4_^+^ cation)
**3**	C20-H20A…Cg1 [1/2 − X, 3/2 − Y, −Z]	2.83	168	3.7748(1)	Cg1: C21-C22-C23-C24-C25-C26 (AsPh_4_^+^ cation) Cg2: C81-C82-C83-C84-C85-C86 (AsPh_4_^+^ cation)
	C22-H22…Cg2 [1/2 − X, 1/2 + Y, 1/2 − Z]	2.99	145	3.7890(1)	

**Table 6:** Selected structural geometric parameters of the coordination polyhedra for **2,2′-bpyPPh_4_** [27], **1** (described as 4,4′-Mebpy), **2,2′-bpyAsPh_4_ [28]**, and **3** (described as 5,5′-Mebpy).

	Angle between Ring Planes in Bipyridyl Ligands (°)	The Shift of the W Atom (Å) in Relation to the Plane Defined Based on Nitrogen and Carbon Atoms in Brackets	The Volume of the Coordination Polyhedron around the W Atom (Å^3^)
**2,2′-bpyPPh_4_** [27]	9.45	0.335 (N7, C12, C13, N8)	17.970
**1**	6.80	0.239 (N1, C6, C7, N12)	18.021
**2,2′-bpyAsPh_4_** [28]	9.77	0.344 (N7, C11, C12, N8)	17.818
**3**	6.20	0.212 (N1, C6, C7, N12)	18.301

**Table 7:** Selected structural parameters for **2,2′-bpyPPh_4_** [27], **1** (described as 4,4′-Mebpy), **2,2′-bpyAsPh_4_ [28]**, and **3** (described as 5,5′-Mebpy).

	2,2′-bpyPPh_4_ [27]	1 (4,4′-Mebpy)	2,2′-bpyAsPh_4_ [28]	3 (5,5′-Mebpy)
volume (Ǻ^3^)	5845.3(1)	3072.11(11)	5725.2(8)	11,915.93(5)
density (mg/m^3^)	1.417	1.409	1.538	1.484
the smallest W-W distance (Ǻ)	9.125	8.864	9.040	9.830
smallest W-W distance between layers (Ǻ)	14.126	13.346	14.034	14.329

## Data Availability

Data are contained within the article.
